# Ozena with Leprosy: United States, 1980

**DOI:** 10.4269/ajtmh.16-0572

**Published:** 2016-10-05

**Authors:** Katherine Murray-Leisure

**Affiliations:** 1 Internal Medicine, Infectious Diseases & Travel Health, Plymouth, Massachusetts. E-mail: kathmurraymd16@comcast.net

Dear Sir:

The three cases of fetid ozena from two Africans and a Saudi Arabian immigrant reported from Minnesota by Yelenich-Huss and others^1^ reminded me of polymicrobial sinus infections among leprosy patients seen in Carville, LA, in the 1980s. *Klebsiella pneumoniae ozaenae* grew mostly as a commensal from the upper respiratory tract of lepromatous leprosy patients with rhinorrhea.^2^ *Klebsiella pneumoniae ozaenae* was found among 16 of 40 or 40% of all *Klebsiella* bacterial isolates from the Gillis Long National Hansen's Disease Center, Carville, LA, in 1980. Carville's leprosy patients then were mostly from North, Central, and South America, Hawaii, or southeast Asia. Among U.S. bloodstream infections reported in 1981 with *K. pneumoniae ozaenae* septicemia and underlying leprosy or *Mycobacterium leprae* infection, Case 2 was a 16-year-old teenage survivor born in the Philippines in 1964.^2^ I suspect today that *K. pneumoniae ozaenae* is a ubiquitous, potentially invasive pathogen present among polymicrobial sinus infections on all continents, including North America.^3^ The *Klebsiella* strain invades the upper respiratory tract of susceptible human hosts around the world. Indeed, among refractory cases of ozena, might one consider adding acid-fast smears to routine nasopharyngeal microbiology evaluations to rule out coinfection with *M. leprae*? Purulent smear-positive, culture-negative acid-fast sinus specimens might prompt acid-fast skin snips or biopsies of the earlobes to exclude early, subclinical lepromatous leprosy.
